# Association rule mining of clinical and biomarker data in neuroendocrine tumors: A prospective study on disease progression

**DOI:** 10.1111/jne.70069

**Published:** 2025-08-14

**Authors:** Ulrich Peter Knigge, Magnus Kjellman, Henning Grønbæk, Espen Thiis‐Evensen, Camilla Schalin‐Jäntti, Staffan Welin, Halfdan Sørbye, Maria del Pilar Schneider, Roger Belusa, The Nordic NET Biomarker Group

**Affiliations:** ^1^ Department of Surgery and Transplantation ENETS Neuroendocrine Tumor Centre of Excellence, Copenhagen University Hospital Copenhagen Denmark; ^2^ Department of Nephrology and Endocrinology ENETS Neuroendocrine Tumor Centre of Excellence, Copenhagen University Hospital Copenhagen Denmark; ^3^ Department of Breast, Endocrine Tumors and Sarcoma Karolinska University Hospital Solna Stockholm Sweden; ^4^ Department of Hepatology and Gastroenterology Aarhus University Hospital, ENETS Center of Excellence Aarhus Denmark; ^5^ Department of Gastroenterology Oslo University Hospital, Rikshospitalet, ENETS Center of Excellence Oslo Norway; ^6^ Endocrinology Abdominal Center, Helsinki University Hospital and University of Helsinki, ENDO‐ERN (European Reference Network on Rare Endocrine Conditions) Helsinki Finland; ^7^ Department of Endocrine Oncology Uppsala University Hospital, ENETS Center of Excellence Uppsala Sweden; ^8^ Cancer Clinic Haukeland University Hospital Bergen Norway; ^9^ Department of Clinical Sciences University of Bergen Bergen Norway; ^10^ Medical Department IPSEN Innovation Paris France; ^11^ Global Biometry Department IPSEN Stockholm Sweden; ^12^ Present address: Morbus Medical AB Stockholm Sweden

**Keywords:** association rules, disease progression, neuroendocrine tumor, plasma chromogranin A, proteins

## Abstract

There is an unmet need for new methods to predict disease course in patients with neuroendocrine tumors (NET). We investigated 92 putative cancer‐related plasma proteins including chromogranin A (CgA) and clinical parameters at the time of diagnosis to identify early factors associated with progressive (PD) or stable disease (SD). Patients with NET grade 1 and 2 of the small intestine (siNET) and pancreas (pNET) were included in this prospective study. Blood samples were obtained at the time of diagnosis before tumor‐related therapy was initiated. During 3 years of follow‐up, SD or PD was determined according to current clinical practice by each investigator. Association rule mining (ARM) was used to identify combinations of biomarkers and clinical parameters associated with SD or PD. Altogether, 115 patients with siNET and 30 with pNET with complete clinical and biomarker data were included in the analysis representing 3 years of follow‐up. Several novel plasma proteins and clinical factors were associated with either PD or SD. In siNET, CgA (>4 upper limits of normal [ULN]) was the most frequent biomarker associated with PD. Females, in contrast to males, with CgA >4 ULN showed a high risk of progression (PPV 100%, NPV 63%). In the siNET cohort, Carboxypeptidase E (CPE) was a discriminating factor between SD and PD. CPE <3.03 was associated with SD, whereas CPE >3.14 was associated with PD (*p* = 0.003). In the pNET cohort, among clinical variables, only the presence of liver metastasis was associated with PD. CgA was not among the top biomarkers associated with PD. Several parameters, both clinical and biomarker data, as well as combinations of these, were associated with PD or SD 3 years after diagnosis. We identified novel biomarkers improving the association with PD or SD. [Correction added on 28 August 2025, after first online publication: Abstract has been updated.]

## 1 INTRODUCTION

Gastroenteropancreatic neuroendocrine tumors (GEP‐NET) are rare tumors with an incidence varying between 2.5 and 5 per 100,000 inhabitants.^1^, ^2^, ^3^, ^4^, ^5^ The majority of GEP‐NET are grade 1 or 2, with a proliferation Ki‐67 index <20%, and are most often slow‐growing tumors.^6^ However, the growth rate differs from patient to patient, and there is an unmet need to identify those patients who have a more rapid growth pattern to enable personalized follow‐up and treatment strategies.

CgA is the single plasma biomarker most used to diagnose GEP‐NET. However, several studies report conflicting results regarding the relationship between CgA levels and treatment response, and its value as a predictor of disease progression has been questioned.^7^, ^8^, ^9^, ^10^ Using a more complex approach, data from studies have demonstrated that a multiple transcript analysis (mRNA obtained from blood) could detect progressive disease at an earlier time point compared with imaging or change in CgA during treatment.^11^, ^12^

Novel biomarkers associated with siNET have been investigated. Edfeldt et al. reported novel proteins that correlated with disease detection as well as survival in 23 patients with siNET, using a proximity ligation assay (PLA) and a proximity extension assay (PEA).^13^ They found three proteins of specific interest: decoy receptor 3 (Dcr3), Trefoil factor 3 (TFF3) and Midkine, also known as neurite growth‐promoting factor 2 (NEGF2). In two previous studies, we investigated machine learning predictive models for novel biomarkers to improve diagnostic accuracy in patients with siNET or pancreatic neuroendocrine tumors (pNET). We demonstrated strong diagnostic accuracy by including the top 10 biomarkers in the predictive model in each cohort.^14^, ^15^, ^16^ Association rule mining (ARM), a well‐known data mining approach, is a rule‐based method for discovering associations between variables in datasets. These algorithms started to be developed in the early 1990s by Agrawal et al.^17^ ARM identifies patterns of frequently occurring events within a dataset, revealing the relationships between them. These patterns highlight combinations of events that tend to occur simultaneously.^18^ Thus, ARM aims to generate new hypotheses and to identify novel relationships. It has been widely used in different fields for pattern mining as well as in the medical field, for example, the identification of medical comorbidities of mental disorders,^19^ factors associated with heart disease in males and females,^20^ drug prescription patterns^21^ and factors associated with loss of control eating in childhood.^22^ It has been used recently to discover symptom patterns in COVID‐19 patients.^18^

The aim of the present study was to use ARM to explore and discover combinations of cancer‐related human plasma proteins, CgA, and clinical factors at the time of diagnosis in patients with siNET or pNET and progressive disease or stable disease within 3 years of follow‐up. In addition, the study aimed to identify and characterize subgroups of patients that have characteristics associated with the risk of disease progression. We hypothesized that the combination of novel proteins and CgA with clinical factors such as Ki‐67%, number of metastases, and symptomatology could provide a better characterization of patients at risk of disease progression, to have a better understanding of the disease and improve disease management.

## 2 METHODS

The present study is the primary endpoint analysis of the EXPLAIN study, including both siNET and pNET patient cohorts for the analysis of disease progression (exploratory, non‐interventional study for evaluating the diagnostic, prognostic and response‐predictive value of a multi biomarker approach in metastatic GEP NET [ClinicalTrials.gov: NCT02630654]). The study was approved by the regional ethical committee in each participating country (Denmark, Finland, Norway, Sweden, Lithuania, Estonia, and Latvia), and the study complied with the Declaration of Helsinki and Good Clinical Practice guidelines. All patients provided written informed consent after a full explanation of the purpose and nature of all procedures used.

### 2.1 Patients

Inclusion criteria: informed consent, suspected metastatic siNET or pNET grade 1 or 2,^23^, ^24^ aged 18 years or older. NET diagnosis was confirmed in all patients according to current clinical practice at each hospital. Five patients with siNET and five with pNET did not have a histological examination. Since this is a non‐interventional study, patients who did not have a histologically confirmed diagnosis at the time of inclusion were kept in the study based on a diagnosis according to current clinical practice. Patients who had surgery of the primary tumor and/or metastases, but with residual disease, could also be included.

Exclusion criteria: NET not confirmed, previous anti‐proliferative treatments, peptide receptor radionuclide therapy (PRRT) or other radiation therapy, other malignant diseases, chronic inflammatory diseases, or severe renal and/or liver disease impairment.

Clinical data were collected at each visit, which occurred at 6‐ to 12‐month intervals and was part of the regular follow‐up. Data was registered in an electronic case report form (VieDoc, Pharma Consulting Group, Uppsala Sweden). Patients using proton pump inhibitors (PPI) were not excluded, nor was the PPI treatment stopped before blood sample collection at patients' normal follow‐up visits. Radiologic evaluation of metastatic disease at the different centers included contrast‐enhanced computed tomography (CT), magnetic resonance imaging (MRI) and somatostatin receptor positron emission tomography (PET)/CT imaging.

Evaluation of disease progression, that is, progressive disease (PD) or stable disease (SD) after 3 years of follow‐up, was evaluated according to clinical practice and investigator judgment using three different criteria (radiological, biochemical and/or clinical) at a multidisciplinary team meeting.

A control group of 155 individuals was selected from the Karolinska University Hospital Clinical Pharmacology Trial Unit for biomarker blood sampling. Control individuals matched by similar age (±5 years) and gender were included in the study (i.e., inclusion criteria). Exclusion criteria included: malignant disease, chronic inflammatory disease, and severe renal or hepatic failure. From this group, a total of 143 individuals were selected after applying the mentioned criteria and with complete biomarker data. Data from the 92 cancer‐related human proteins were used to calculate mean 95% confidence intervals (CIs) to define cut‐off values for biomarker categorization in siNET and pNET for association rules mining.

### 2.2 Sample collection and biomarker analysis

Blood sample (4 mL) was collected starting at the first visit before NET specific treatment was initiated for siNET and pNET as well in the control group. Thereafter, blood samples were collected at patients' regular follow‐up visits. Details on sample collection and laboratory biomarker analysis are provided in the Supporting Information.

### 2.3 Statistical analysis

#### 2.3.1 Association rule mining

Association rule mining was performed by means of the a priori *algorithm* ^25^ implemented in the R package *arules*.^26^ Association rules were derived independently in siNET and pNET cohorts by including CgA, 92 cancer‐related human proteins (herein called “biomarkers”) and clinical characteristics (Table S1) evaluated at the time of diagnosis to explore and discover associations between the mentioned variables with disease progression.

ARM was used to detect patterns of frequent items or events in the data set, including the association between items or events that occur at the same time. An association rule was defined as an implication of the form *X* = >*Y*, where X,Y⊆I and X∩Y=0. *X* and *Y* were called antecedent (left‐hand‐side [LHS]) and consequent (right‐hand‐side [RHS]) of the rule, respectively. This relationship identified the simultaneous occurrence between items in antecedent *X* and consequent *Y*.

ARM generated association rules (LHS → RHS) that were evaluated and selected using the following metrics:

- *Support*: the number of times that the rule was found in the dataset; how often the joined rule LHS and rule RHS occurred among all the groups.
- *Confidence*: the measure of how frequently the rule RHS occurred among all the groups containing the rule LHS. The confidence value indicates how reliable the rule was.
- *Lift*: the confidence of the rule divided by the expected confidence, assuming that *X* and *Y* are independent. This is often used to measure the interest of, or the importance of a rule. Lift measures the performance of a rule to identify a subgroup from a larger population. Rules with a lift value greater than 1 provide a positive correlation, whereas rules with a lift value less than 1 provide a negative correlation. A lift value close to 1 indicates *X* and *Y* are independent. An example is provided in the Supporting Information.

#### 2.3.2 Variable categorization

ARM requires numerical variables (e.g., biomarkers, age) to be transformed into categories. Variable categorization can be based on data distribution (e.g., mean or median values) or by using relevant clinical cut‐off values (e.g., biomarker level, specific age associated with disease onset). It is a step commonly applied by logical approaches to detect non‐linear relations between variables.^27^ Categorization strategies applied for variables included in ARM, that is, CgA, 92 biomarkers and clinical variables, are provided in the Supporting Information and Table S1.

#### 2.3.3 Association rules

ARM was used to generate association rules for disease progression (SD and PD) independently in siNET and pNET. Up to three variables (i.e., attributes) could be combined in the LHS of the rule (i.e., CgA, 92 biomarkers and clinical variables). After rules generation, only rules containing disease progression information (i.e., SD or PD) in the right‐hand side of the rule were selected and analyzed. To select the most relevant rules associated with disease progression, minimum cut‐off values were defined for lift, support, and confidence. For support, a minimum value was set allowing a rule to occur in at least 30% of patients. For confidence and lift, minimum values were set at 70% and 1.2, respectively, for both cohorts. Redundant rules, that is, rules that have the same attributes but in a different order (e.g., female ∩ biomarker Z → PD or biomarker Z ∩ female → PD), were removed, given that they do not provide additional information. ARM analyses were carried out using R 4.1.0.^28^

#### 2.3.4 Statistical test

Statistical tests were performed to evaluate the effect of treatment interventions on disease progression (i.e., nominal logistic regression). Analysis of variance (ANOVA) was performed to test the effect of disease progression on carboxypeptidase E (CPE). The ANOVA model included treatment effect. Statistical tests were performed using JMP PRO, version 17.0.^29^

## 3 RESULTS

### 3.1 Clinical characteristics evaluated at time of diagnosis

Patients who did not fulfill the inclusion/exclusion criteria or had incomplete data regarding disease progression or missing biomarker data were excluded from the ARM analysis, leaving 145 patients, of which 115 were diagnosed with siNET and 30 had pNET. After 3 years of follow‐up, 65 (57%) patients with siNET and 22 (73%) with pNET had progressed. Eighty‐two percent (53/65) of siNET and 100% (22/22) of pNET patients with progressive disease had radiological confirmation of one or more additional metastases or clinically significant growth of one or more metastases. In the remaining siNET patients, progression was determined according to clinical or biochemical progression. Eight (6.9%) siNET and 2 (6.7%) pNET patients died during follow‐up and were included in the PD groups. Clinical characteristics for both siNET and pNET patients are given in Table 1.

**TABLE 1:** Clinical characteristics at time of diagnosis in patients with siNET and pNET with stable or progressive disease within 3 years of follow‐up.

Clinical characteristic	siNET		pNET	
	SD (*n* = 50)	PD (*n* = 65)	SD (*n* = 8)	PD (*n* = 22)
Age (years)				
Median (range)	67 (38–88)	66 (41–82)	65 (53–77)	68 (46–82)
Gender, *n* (%)				
Female	15 (30%)	32 (49%)	4 (50%)	11 (50%)
Lymph node metastasis, *n* (%)				
N0	10 (20%)	13 (20%)	5 (63%)	8 (36%)
N1	40 (80%)	51 (78%)	3 (38%)	14 (64%)
Nx	0 (0%)	1 (2%)	0 (0%)	0 (0%)
Distant metastasis, *n* (%)				
M0	16 (25%)	5 (8%)	2 (25%)	1 (5%)
M1	34 (52%)	60 (92%)	6 (75%)	21 (95%)
Mx	0 (0%)	0 (0%)	0 (0%)	0 (0%)
NET grade, *n* (%)				
G1	28 (56%)	18 (28%)	4 (50%)	5 (23%)
G2	20 (40%)	43 (66%)	3 (38%)	13 (59%)
Missing data	2 (4%)	4 (6%)	1 (12%)	4 (18%)

Abbreviations: G1, grade 1 NET classification; G2, grade 2 NET classification; PD, progressive disease; pNET, pancreatic NET; SD, stable disease; siNET, small‐intestinal NET.

### 3.2 Treatment interventions during the study

NET‐related treatments that were initiated after disease diagnosis and followed according to study protocol are presented in Table 2. Patients were treated according to the decision of the physician following current medical guidelines and available treatment options and/or tumor board decisions. The difference in treatment options did not have a statistically significant effect on disease progression, either in siNET (*p* = 0.10) or in pNET (*p* = 0.35).

**TABLE 2:** Treatment interventions initiated within 9 months after disease diagnosis in patients with siNET and pNET.

	siNET		pNET	
	PD (*n* = 65)	SD (*n* = 50)	PD (*n* = 22)	SD (*n* = 8)
Treatment, *n* (%)				
SSA	37 (57%)	21 (42%)	16 (76%)	4 (57%)
SSA and surgery	12 (18%)	18 (36%)		
Other treatments^a^	16 (25%)	11 (22%)	5 (24%)	3 (43%)
Missing			1 (0%)	1 (0%)

Abbreviations: PD, progressive disease; pNET, pancreatic NET; SD, stable disease; siNET, small‐intestinal NET; SSA, somatostatin analogues.

^a^ Peptide receptor radionuclide therapy (PRRT), chemotherapy, mTOR inhibitor, interferon‐alpha and/or liver artery embolization.

### 3.3 Association rule mining: Evaluation of disease progression in patients with siNET

Exploration of variables was evaluated at the time of diagnosis and disease progression in the siNET group using ARM. A total of 2231 rules associated with PD were selected for siNET after applying the following filtering: support >20, lift ≥1.2, and confidence ≥70%. Among the selected rules, the top 5 rules ranked by highest lift and support are presented in Table 3. Overall, combinations between biomarkers and clinical variables (rule LHS) were associated with PD (rule RHS). The most frequent biomarkers and clinical variables associated with PD in 2231 rules are shown in Table S3. CgA >4 ULN was the most frequent single biomarker associated with PD. Other novel biomarkers identified to be associated with PD were WISP‐1 >3.73, vimentin (VIM) >1.74, and carboxypeptidase E (CPE) >3.14. Among the clinical variables, the most frequent characteristics associated with PD were NET grade 2, presence of liver metastasis, having more than 10 metastases, and having carcinoid syndrome (CS) symptoms (i.e., diarrhea, flushing, bronchospasm).

**TABLE 3:** Top 5 rules ranked by lift and support associated with stable and progressive disease in patients with siNET.

Rule LHS	Rule RHS	Support	Confidence	Lift
CgA > 4 UNL ∩ Ki‐67 > 5% ∩ WISP‐1 > 3.73	PD	26	1.00	2.08
NET grade 2 ∩ CgA > 4 UNL ∩ TFPI‐2 > 7.07	PD	25	1.00	2.08
CgA > 4 UNL ∩ Ki‐67 > 5% ∩ ABL1 > 2.04	PD	24	1.00	2.08
Female ∩ CgA > 4 UNL	PD	23	1.00	2.08
Ki‐67 > 5% ∩ liver metastasis ∩ CPE > 3.14	PD	21	1.00	2.08
CPE <3.03 ∩ PVRL4 > 5.38 ∩ ADAM‐8 > 3.90	SD	15	0.94	2.53
No CS symptoms ∩ TGF‐alpha > 1.21 ∩ CD160 > 4.52	SD	15	0.88	2.38
No CS symptoms ∩ MK > 5.90 ∩ CD160 > 4.52	SD	15	0.88	2.38
NET grade 1 ∩ No diarrhea CS symptom ∩ PPY > 5.42	SD	15	0.88	2.38
CPE < 3.03 ∩ IFN‐gamma‐R1 > 3.28 ∩ MIA > 9.52	SD	15	0.88	2.38

*Note*: Biomarkers: ABL1, tyrosine‐protein kinase; ADAM8, disintegrin and metalloproteinase domain‐containing protein; CD160, CD160 antigen; CPE, carboxypeptidase E; IFN‐gamma‐R1, interferon gamma receptor 1; MIA, melanoma‐derived growth regulatory protein; MK, midkine; PPY, pancreatic prohormone; PVRL4, nectin‐4; TFPI‐2, tissue factor pathway inhibitor 2; TGF‐alpha, transforming growth factor alpha; WISP‐1, WNT1‐inducible‐signaling pathway protein 1. Description of abbreviations for biomarkers is presented at https://olink.com/products‐services/target/oncology‐panel/ (accessed 5 December 2024).

Abbreviations: CgA, chromogranin A; CS, carcinoid syndrome symptoms: diarrhea, flushing, bronchospasms; LHS, left hand side; PD, progressive disease; RHS, right hand side; SD, stable disease; ULN, upper limit of normal; ∩, intersection.

**FIGURE 1:**
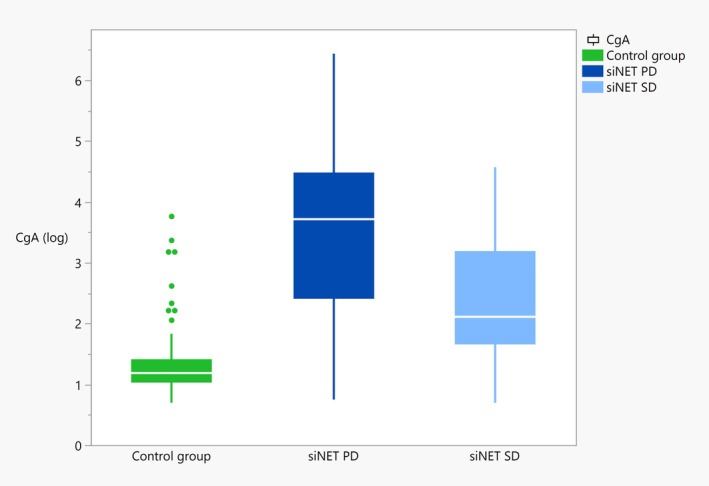
Levels of CgA (A) and CPE (B) in patients with siNET by disease status and control group. CgA, chromogranin A in log scale; CPE, carboxypeptidase E; PD, progressive disease; SD, stable disease; siNET, small‐intestinal NET. Control group: 143 individuals; siNET SD: 50 patients; siNET PD: 65 patients. Descriptive statistics: mean (SD) [95% confidence interval]: (A) CgA (in log scale): control group: 1.29 (0.48) [1.21–1.37]; siNET PD: 3.57 (1.37) [3.24–3.91]; siNET SD: 2.35 (1.01) [2.06–2.63]. (B) CPE: control group: 3.09 (0.34) [3.03–3.14]; siNET PD: 3.32 (0.74) [3.13–3.50]; siNET SD: 2.96 (0.33) [2.87–3.06].

Among the rules associated with PD in siNET (Table 3), females with CgA >4 ULN at the time of diagnosis had a two times (lift = 2.08) higher risk of PD within 3 years of follow‐up. This subgroup corresponded to 23 out of 32 females (72%) with PD. It corresponds to a positive predictive value (PPV) of 100% and a negative predictive value (NPV) of 63%. In males, these values were 62% and 65%, respectively. Furthermore, to better characterize the subgroup associated with females, new association rules were generated by selecting in the RHS the combination of females with a CgA >4 ULN. Thus, 54 rules were selected with a support ≥8, confidence ≥80%, and characterized by a very high lift (>4.7). Top rules are presented in Table S3. Among the 54 selected rules, the most frequent variable associated with females with CgA >4 ULN and PD can be mentioned: 5‐HIAA >455 (% ULN), Ki‐67 >5%, CPE >3.14, and more than 10 total metastases. Figure 1A displays the CgA levels for siNET disease status, including the control group.

For SD in siNET, a total of 216 association rules were identified with a lift ≥1.2, confidence ≥70%, and support >14. The top 5 rules ranked by highest lift and support are shown in Table 3. The first rule showed that patients with a level of CPE <3.03 and PVRL4 >5.38 and ADAM‐8 >3.90 had 2.5 times (lift = 2.53) higher risk of being associated with SD. The most frequent biomarkers and clinical variables identified in 216 rules that were associated with SD are presented in Table S2. The most frequent biomarkers associated with SD were CPE <3.03, DC160 >4.52, and PVRL4 >5.38. CgA was not found among the 10 most frequent biomarkers associated with SD. Overall, patients with NET grade 1 and no CS symptoms of diarrhea were associated with SD.

CPE was associated with both PD and SD. Thus, CPE <3.03 was associated with SD, whereas CPE >3.14 was associated with PD. Statistically significant differences (*p* = 0.03) were found for CPE levels between SD and PD (least square mean (standard error) [lower and upper 95% CI]), that is, PD: 3.32 (0.08) [3.17–3.48]; SD: 2.99 (0.09) [2.83–3.16]. CPE levels by siNET disease status and including the control group are shown in Figure 1B. For illustration, receiver operating characteristic (ROC) curves and area under the curve (AUC) for CgA, CPE, WISP‐1, and CD160 are shown in Figure S2.

### 3.4 Association rule mining: Evaluation of disease progression in patients with pNET

Exploration of variables evaluated at the time of diagnosis and disease progression was performed on pNET using ARM. Rules associated with SD are not shown due to the small size of the group.

A total of 29 rules associated with PD were identified for patients with pNET, after applying the following filters: support >14, lift ≥1.25, and confidence ≥70%. The top 5 rules associated with PD, ranked by lift and support, are presented in Table 4. Mainly, biomarkers were found to be associated with PD. Among clinical characteristics, only the presence of liver metastasis was associated with PD. The most frequent biomarkers associated with PD in 29 rules were TRAIL <7.27, DLL1 >8.79, and ITGAV <2.72 (Table S4). CgA was not identified in the list of most frequent parameters associated with PD.

**TABLE 4:** Top 5 rules ranked by lift and support associated with PD in patients with pNET.

Rule LHS	Rule RHS	Support	Confidence	Lift
5′‐NT > 9.56 ∩ DLL1 > 8.79 ∩ VIM > 1.74	PD	15	1.00	1.36
5′‐NT > 9.56 ∩ DLL1 > 8.79	PD	16	0.94	1.28
Liver metastasis ∩ ITGAV < 2.72 ∩ TRAIL < 7.27	PD	15	0.94	1.28
ITGAV < 2.72 ∩ TRAIL < 7.27 ∩ ICOSLG < 3.21	PD	15	0.94	1.28
ITGAV < 2.72 ∩ TRAIL < 7.27 ∩ VEGFR‐2 < 6.32	PD	15	0.94	1.28

*Note*: Biomarkers: 5'‐NT, 5'‐nucleotidase; DLL11, delta‐like protein 1; ICOSLG, ICOS ligand; ITGAV, integrin alpha‐V; TRAIL, TNF‐related apoptosis‐inducing ligand; VEGFR‐2, vascular endothelial growth factor receptor 2; VIM, vimentin. Description of abbreviations for biomarkers is presented at https://olink.com/products‐services/target/oncology‐panel/ (accessed 5 December 2024).

Abbreviations: LHS, left hand side; PD, progressive disease; RHS, right hand side; ∩, intersection.

## 4 DISCUSSION

This is the first prospective study to use an ARM data mining approach to characterize progressive disease within 3 years from diagnosis in GEP‐NET patients, including 92 cancer‐related plasma proteins, including CgA and clinical characteristics at the time of diagnosis, before NET‐specific treatment intervention. The main finding in this exploratory study was that ARM identified both novel biomarkers and combinations with clinical characteristics associated with stable or progressive disease. The most important parameters at the time of diagnosis associated with disease progression were CgA, Ki 67%, gender, and the novel biomarker CPE.

In patients with siNET, high levels of CgA and higher Ki 67% in combination with CPE or ABL1 at the time of diagnosis were strongly associated with PD. Since CPE is involved in peptide processing in neuroendocrine cells, the level of this biomarker could reflect the number and/or activity of these cells. Previous studies have linked expression of CPE to growth and metastatic behavior in several cancers.^30^, ^31^ Recently, CPE was reported as a novel marker for the identification of pancreatic neuroendocrine tumors.^32^ The predictive value of CPE levels in GEP‐NET needs further validation. Additional clinical parameters evaluated at baseline and associated with PD were NET grade 2, liver metastasis, having more than 10 metastases, and the presence of carcinoid‐like symptoms.

Differences in cancer outcomes between genders is a known factor^33^ and male sex is associated with worse outcomes in NEN patients.^34^ In our study among patients with siNET, females with high levels of CgA (>4 ULN) at the time of diagnosis were at a higher risk of disease progression. Gender differences in SSA efficacy have recently been addressed in a review; however, in most studies, gender was not a predictor of response to treatment.^35^ Further investigation whether gender differences relate to disease progression in NET is of value.

Novel biomarkers associated with SD in siNET were low CPE and high CD160 (Tables 3 and S2, Figure S1). This association was stronger in patients lacking carcinoid‐like symptoms. CD160 is an immune checkpoint inhibitor and co‐inhibitory receptor expressed on T cells,^36^ where increased plasma levels may indicate high immune activity. An increased level of CPE (>3.14) in plasma was associated with PD, while a lower level (<3.03) was associated with SD. Low levels of CgA at the time of diagnosis were not among the biomarkers associated with SD. In a large prospective study with 239 patients, Dam et al. showed only a weak association between change in plasma CgA and change in tumor burden in a cohort of GEP‐NET patients, suggesting that CgA as a single biomarker is inadequate to predict tumor progression.^10^ However, in the RADIANT‐2 study, a baseline serum CgA level of >10× ULN was a predictive factor for shorter time to radiological progression.^37^ In addition, patients with SD were characterized at the time of diagnosis by having NET grade 1, absence of flushing, and abdominal pain except for the presence of diarrhea. These clinical parameters are included in known NET nomograms associated with disease prognostic.^38^, ^39^, ^40^

Elevated VIM levels are frequently associated with PD in pNET (as well in siNET).^41^ VIM is a protein involved in cellular motility, cell shape maintenance, and the direction of cell migration, and high VIM expression, with loss of E‐cadherin expression, is correlated with disease progression and a poor prognosis in patients with grade 1 and 2 pNET following resection.^41^ Molecular profiling of pNET showed a strong up‐regulation of VIM^42^ and a strong positive reaction to staining for VIM has been identified in Pnet.^43^ Moreover, aberrant expression of VIM is reported in lung carcinoid tumours.^44^ WISP‐1 (also known as CCN4) is also associated with PD; like other CCNs, it plays multiple physiologic roles in development and participates in disease pathogenesis. CCN4 is of particular interest in cancer, as it shows promise as a biomarker or prognostic factor as well as a potential therapeutic target.^45^

Regarding novel biomarkers, low levels of TNF‐related apoptosis‐inducing ligand (TRAIL) and high levels of delta‐like protein 1 (DLL1) were the most frequent biomarkers associated with PD (Table 4). DLL1 has been shown to be involved in cell differentiation and proliferation, and TRAIL has been linked to apoptosis.^46^, ^47^

In contrast to siNET, CgA was not among the most frequent biomarkers in the selected rules associated with PD in pNET. In the RADIANT‐3 study, CgA levels >2 ULN only showed a weak association with shorter progression‐free survival (HR 1.33 [95% CI 0.98–1.82, *p* = 0.035]).^48^ In a large retrospective study, the combination of CgA and plasma cocaine‐ and amphetamine‐regulated transcript (CART) levels in the blood indicated progressive disease in patients with pNET.^49^

The current exploratory study has several strengths which contribute to its value: prospective design, real‐world clinical setting, large siNET patient cohort, centralized biomarker analysis, and the use of data‐driven statistical methods. A key strength of the study is its prospective design, focusing on disease progression within 3 years of diagnosis. However, its non‐interventional nature, intended to mimic routine clinical practice, limited the ability to collect additional blood samples, which is an important limitation. In contrast, this mirrors the real‐life situation which is of value to underscore its applicability.

Disease progression was not assessed using RECIST criteria.^50^ It was evaluated and determined according to routine clinical practice (e.g., radiological, biochemical, and/or symptomatic changes), followed by a change in treatment. Although more than 80% of progression assessments were based on radiological evidence, the lack of standardized criteria such as RECIST remains a limitation. This may have reduced the precision in distinguishing between progression and non‐progression, potentially leading to falsely low sensitivity and specificity in the model. Patients were treated according to the decision of the physician, following current medical guidelines and available treatment options and/or tumor board decisions. Most patients were treated with SSA after diagnosis, which could have impacted disease outcome. To improve accuracy, future studies should apply RECIST criteria for the classification of disease progression. Nevertheless, the majority of patients in the progression cohort did show radiologically confirmed increases in the number or size of metastases on routine imaging.

ARM is a known data mining technique used in many fields, and it is part of the “interpretable” machine learning methods, which can be useful in the context of analyzing and interpreting medical data. However, ARM requires categorization of data, which might result in a loss of information, and cut‐off values defined for categorization could be considered arbitrary.

Plasma protein levels are the results of both disease status as well as combinations of other unknown factors at the time of diagnosis, and it provides an opportunity to evaluate complex disease situations. Increasing the number of plasma proteins analyzed (e.g., a larger protein panel) could help to identify protein pathways associated with the disease and to improve the accuracy of the predictions. Further studies are needed to evaluate the place of these novel biomarkers in disease development and management. It would be of great interest to further analyze and validate novel biomarkers using immuno‐assay techniques.

In conclusion, at diagnosis ARM identified novel biomarkers, alone or in combination with clinical characteristics, associated with SD and PD over the next 3 years. The application of data‐driven approaches, such as ARM, may bring value to identify relevant risk factors and novel biomarkers or combinations at time of diagnosis that could be associated with the disease course, and thus enable physicians to adjust treatment strategy and frequency of follow‐up accordingly.

## AUTHOR CONTRIBUTIONS

**Ulrich Peter Knigge:** Conceptualization; investigation; writing – original draft; writing – review and editing; supervision; methodology; validation. **Magnus Kjellman:** Conceptualization; investigation; writing – original draft; writing – review and editing; supervision; methodology; validation. **Henning Grønbæk:** Conceptualization; investigation; writing – original draft; writing – review and editing; methodology; supervision; validation. **Espen Thiis‐Evensen:** Conceptualization; investigation; writing – original draft; writing – review and editing; supervision; methodology; validation. **Camilla Schalin‐Jäntti:** Conceptualization; investigation; writing – original draft; writing – review and editing; supervision; methodology; validation. **Staffan Welin:** Conceptualization; writing – original draft; writing – review and editing; investigation; methodology; supervision; validation. **Halfdan Sørbye:** Conceptualization; investigation; writing – original draft; writing – review and editing; methodology; supervision; validation. **Maria del Pilar Schneider:** Writing – original draft; writing – review and editing; visualization; methodology; formal analysis; software; data curation; validation. **Roger Belusa:** Conceptualization; investigation; writing – original draft; writing – review and editing; methodology; validation; supervision; data curation; formal analysis; project administration.

## FUNDING INFORMATION

This study was funded by IPSEN.

## CONFLICT OF INTEREST STATEMENT

Halfdan Sørbye: Consultant/advisory board: BMS, Advanced Accelerator, IPSEN; Lecture honoraria: IPSEN, SAM Nordic. Maria del Pilar Schneider: Employee of IPSEN and hold stock options at IPSEN. All other authors declare no conflicts of interest.

## PEER REVIEW

The peer review history for this article is available at https://www.webofscience.com/api/gateway/wos/peer-review/10.1111/jne.70069.

## ETHICS STATEMENT

This study was approved by the local Ethics Committees of the Nordic countries, and written informed consent was obtained from all patients.

## Supporting information


**Data S1.** Supporting Information.

## Data Availability

The data that support the findings of this study are available from the corresponding author upon reasonable request.
