# Supplementary material for: Association rule mining of clinical and biomarker data in neuroendocrine tumors: A prospective study on disease progression
Source: J Neuroendocrinol. 2025 Aug 14;37(10):e70069. doi: 10.1111/jne.70069 (PMC12488364; doi:10.1111/jne.70069)
Supplement: Supplementary file 1 — Data S1. Supporting Information. [file JNE-37-e70069-s001.docx]

**Supplementary Materials**

**Sample collection and biomarker analysis**

Blood sample (4 ml) was collected starting at the first visit before any NET specific treatment was initiated for siNET and pNET as well in the control group. Thereafter blood samples were collected at patients’ normal follow-up visits. Blood samples were collected in tubes containing ethylenediaminetetraacetic acid and placed on ice immediately after sampling. The samples were centrifuged at 2500 g for 10 min at 4°C. Thereafter plasma was aspirated, aliquoted to new tubes (4 x 0.5 ml) and immediately stored at −80°C. Blood samples for the exploratory plasma protein biomarkers were transported on dry ice to SciLifeLab Uppsala, Sweden, and analyzed using multiplex proximity extension assay (PEA) by real-time PCR using the Fluidigm BioMark HD real-time PCR platform, with the Olink Proseek Oncology II panel (Olink Proteomics, Uppsala, Sweden, http://www.olink.com/) as previously described [^24,25^](https://paperpile.com/c/0bgY2w/b0HYv+0b7As). The multiplex PEA was developed from the proximity ligation assay (PLA) technique by Lundberg et al. 2011[^26^](https://paperpile.com/c/0bgY2w/eXou). One pair of antibodies was used to target each specific protein. The antibodies were coupled to complementary oligonucleotides enabling DNA polymerase to amplify the double-stranded DNA, generating a PCR-reporter sequence by the proximity-dependent DNA polymerization event. The proteins in the Olink Proseek Oncology II panel were selected as they previously had been shown to be associated with neoplastic disease and classified according to Uniprot, Human Protein Atlas, Gene Ontology and DisGeNET[^27,28^](https://paperpile.com/c/0bgY2w/waCbm+pNKaq). Description of abbreviations for biomarkers is presented at [Olink Target 96 — Olink®](https://olink.com/products/olink-target-96) (Accessed 05 December 2024).

CgA was analyzed centrally at Akademiska Laboratoriet Uppsala, Sweden with the NEOLISA™ Chromogranin A assay (Euro Diagnostica). Urine/serum 5-HIAA was analyzed at each individual hospital using methods according to clinical routine and presented as percent of upper limit of normal (% ULN). Serum 5-HIAA was measured at 3 clinics in Finland, morning urine 5-HIAA was used in one clinic in Norway and remaining clinics used 24-hour urine samples. Ki-67 was analyzed according to each hospital's routine clinical practice.

**Association rules metrics example**

**
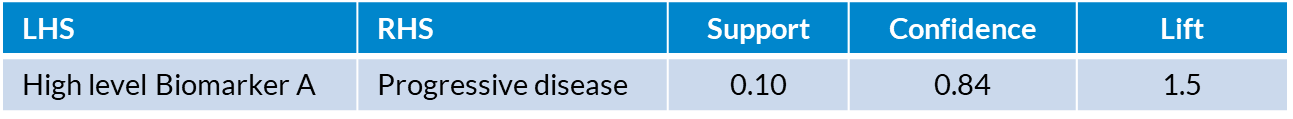
**

**Support:** The support of having progressive disease when biomarker A is high is 11%

**Confidence:** It is how likely disease progression occur in patients who have high levels of biomarker A. Thus, the confidence of having progressive disease when biomarker level A is high is **84%.**

**Lift:** Lift measures how likely disease progression occurs in patients with high levels of biomarker A controlling for disease progression Thus, there is **1.5** more probability to have progressive disease when patients have high levels of biomarker A.

**Variable categorization**

Association rule mining (ARM) requires numerical variables (e.g., biomarkers, age) to be transformed into categories. Variable categorization can be based on data distribution (e.g. using the mean or median values, or cut-off thresholds) or by using relevant clinical cut-off values (e.g. specific age associated with disease onset). It is a step commonly applied by logical approaches to detect non-linear relations between variables (Tufféry et al., 2011).

The following steps were applied for the categorization of 92 biomarkers:

- The 95% mean confidence intervals (CI) were calculated for each biomarker from the control group and thus for each biomarker the lower (L) 95% CI and upper (U) 95% CI were defined as cut-off values for categories definition.
- Then, for each siNET or pNET patient, each biomarker value was categorized into three groups by using as cut-off values calculated from the control group (i.e. L 95% CI and U 95% CI) and labeled as:
  - *Low:* biomarker value *i* < L 95% CI of the corresponding biomarker in the control group;
  - *Normal:* biomarker value *i* ≥ L 95% CI and *i* ≤ U 95% CI of the corresponding biomarker in the control group; and
  - *High:* biomarker value *i* > U 95% CI of the corresponding biomarker in the control group

For CgA, upper limit normal (ULN) was used as categorization cut-off value. Categorized CgA variable was labelled as:

- Normal: CgA ≤ 5.3 nmol/L,
- High: CgA > 5.3 nmol/L and CgA < 4 * 5.3 nmol/L,
- Very high: CgA > 4 * 5.3 nmol/L (i.e., 21.2 nmol/L).

The CgA value of 5.3 nmol/L is 97.7th percentile mean value calculated from the control group.

Clinical parameters included in ARM and corresponding categorization are presented in Table S1.

Table S1: Variables evaluated at time of diagnosis with corresponding categorization strategy.

| **Variables** | **Number of categories** | **Category label** | **Description** |
| --- | --- | --- | --- |
| Gender | 2 | Female/male |  |
| Age group (years) | 3 | < 61, 61-69, >69 | Derived age groups |
| Surgery prior inclusion | 2 | Yes/No | Surgery of primary tumor prior inclusion |
| NET Grade | 2 | Grade 1, Grade 2 | Grade defined according ENETS Guidelines & Standards of Care |
| Ki-67% | 3 | <=1.0%, > 1.0% & <=5.0%, > 5.0% | Derived |
| 5-HIAA | 3 | <108.0, >=108.0 & <=455.3, >455.3 | Derived (i.e. discretized using tiertiles) |
| Carcinoid Syndrome |  |  |  |
| *Symptom diarrhea* | 2 | Yes/No | Presence of symptom |
| *Symptom abdominal pain* | 2 | Yes/No | Presence of symptom |
| *Symptom flushing* | 2 | Yes/No | Presence of symptom |
| *All Symptoms* | 2 | Yes/No | Derived. To account whether a patient presents the three Carcinoid Syndrome symptoms |
| Metastasis |  |  |  |
| *Liver Metastasis* | 2 | Yes/No | Presence of metastases |
| *Lymph Metastasis* | 2 | Yes/No | Presence of metastases |
| *Other Metastasis* | 2 | Yes/No | Presence of metastases |
| *Total Metastasis* | 3 | <=2, 3 to 9, >=10 | Derived. Total number of metastases |
| *CgA* | 3 | *Normal:* CgA <= 5.3 nmol/L *High*: CgA >5.3 nmol/L & CgA <= 21.2 nmol/L *Very high: CgA* > 4 ULN (5.3nmol/L, i.e. 21.2 nmol/L) | Derived |
| *Plasma protein* | 3 | *Low:* biomarker value < L 95% CI of the corresponding biomarker in the control group *Normal:* biomarker value ≥ L 95% CI and ≤ U 95% CI of the corresponding biomarker in the control group biomarker value  *High:* biomarker value > U 95% CI of the corresponding biomarker in the control group | Derived for the 92 plasma proteins |

Table S2: Most frequent biomarkers and clinical characteristics identified in 2,231 rules associated with progressive disease and 216 rules associated with stable disease in patients with siNET.

| **Biomarkers** | **Count, n (%)** |  | **Clinical characteristics and biomarkers** | **Count, n (%)** |
| --- | --- | --- | --- | --- |
| *Associated with PD* |  |  | *Associated with PD* |  |
| CgA > 4 ULN | 683 (10.5%) |  | CgA > 4 ULN | 683 (10.5%) |
| WISP-1 > 3.73 | 189 (2.9%) |  | NET Grade 2 | 665 (10.2%) |
| VIM > 1.74 | 132 (2.0%) |  | Liver metastasis | 578 (8.9%) |
| CPE > 3.14 | 121 (1.9%) |  | WISP-1 > 3.73 | 189 (2.9%) |
| MK > 5.90 | 116 (1.8%) |  | More than 10 metastases | 179 (2.8%) |
| TXLNA > 2.64 | 114 (1.8%) |  | All Carcinoid Syndrome symptoms | 170 (2.6%) |
| CDKN1A > 2.51 | 111 (1.7%) |  | Ki-67 > 5% | 156 (2.4%) |
| TNFSF13 > 7.94 | 104 (1.6%) |  | No surgery of primary tumor prior inclusion | 150 (2.3%) |
| TFPI-2 > 7.07 | 99 (1.5%) |  | VIM > 1.74 | 132 (2.0%) |
| LYN > 0.96 | 93 (1.4%) |  | CPE > 3.14 | 121 (1.9%) |
|  |  |  |  |  |
| *Associated with SD* |  |  | *Associated with SD* |  |
| CPE < 3.03 | 57 (8.9%) |  | CPE < 3.03 | 57 (8.9%) |
| CD160 > 4.52 | 46 (7.1%) |  | NET Grade 1 | 56 (8.7%) |
| PVRL4 > 5.38 | 33 (5.1%) |  | CD160 > 4.52 | 46 (7.1%) |
| MIA > 9.52 | 33 (5.1%) |  | No CS symptom of diarrhea | 34 (5.3%) |
| SCF > 8.40 | 21 (3.3%) |  | PVRL4 > 5.38 | 33 (5.1%) |
| ADAM-TS-15 > 3.71 | 21 (3.3%) |  | MIA > 9.52 | 33 (5.1%) |
| TNFRSF19 > 3.68 | 18 (2.8%) |  | ADAM-TS-15 > 3.71 | 21 (3.3%) |
| MK > 5.90 | 13 (2.0%) |  | Males | 21 (3.3%) |
| ADAM-8 > 3.90 | 12 (1.9%) |  | SCF > 8.40 | 21 (3.3%) |
| ITGAV < 2.72 | 12 (1.9%) |  | No other metastasis | 20 (3.1%) |

CS: Carcinoid syndrome symptoms: diarrhea, flushing or abdominal pain; PD: progressive disease; SD: stable disease; siNET: small-intestinal NET; ULN: Upper Limit of Normal. Description of abbreviations for biomarkers is presented at <https://olink.com/products-services/target/oncology-panel/> and [Olink Target 96 — Olink®](https://olink.com/products/olink-target-96) (Accessed 05 December 2024)

Table S3: Top 5 rules ranked by lift and support associated with females siNET patients with CgA > 4 ULN and progressive disease.

| **Rule LHS** | **Rule RHS** | **Support** | **Confidence** | **Lift** |
| --- | --- | --- | --- | --- |
| Ki-67 > 5% ∩ CPE > 3.14 ∩ KLK13 < 4.36 | Female ∩ CgA > 4 ULN | 11 | 0.79 | 4.61 |
| Ki-67 > 5% ∩ ESM-1 > 8.18 ∩ MAD-homolog-5 < 2.93 | Female ∩ CgA > 4 ULN | 10 | 0.77 | 4.52 |
| CD27 < 7.16 ∩ WISP-1 > 3.62 ∩ GZMB < 2.18 | Female ∩ CgA > 4 ULN | 10 | 0.77 | 4.52 |
| TFPI-2 >7.07 ∩ FASLG < 8.12 ∩ MAD-homolog-5 < 2.93 | Female ∩ CgA > 4 ULN | 10 | 0.77 | 4.52 |
| Ki-67 > 5% ∩ CPE > 3.14 ∩ TRAIL < 7.27 | Female ∩ CgA > 4 ULN | 11 | 0.73 | 4.30 |
| Total metastases > 10 ∩ CPE > 3.14 ∩ KLK13 < 4.36 | Female ∩ CgA > 4 ULN | 10 | 0.71 | 4.19 |

CgA: Chromogranin A; siNET: small intestinal NET; ULN: Upper Limit of Normal; ∩: intersection. Description of abbreviations for biomarkers is presented at [Olink Target 96 — Olink®](https://olink.com/products/olink-target-96) (Accessed 05 December 2024)

Table S4: Most frequent biomarkers identified in 29 rules associated with progressive disease in patients with pNET.

| **Biomarkers associated with PD** | **Count (%)** |
| --- | --- |
| TRAIL < 7.27 | 12 (15.4%) |
| DLL1 > 8.79 | 10 (12.8%) |
| ITGAV < 2.72 | 8 (10.3%) |
| VIM > 1.74 | 8 (10.3%) |
| 5'-NT > 9.56 | 7 (9.0%) |

PD: progressive disease; pNET: pancreatic NET. Description of abbreviations for biomarkers is presented at <https://olink.com/products-services/target/oncology-panel/> and [Olink Target 96 — Olink®](https://olink.com/products/olink-target-96) (Accessed 05 December 2024)

Figure S1. Boxplot of CD160 levels in patients with siNET by disease status and control group


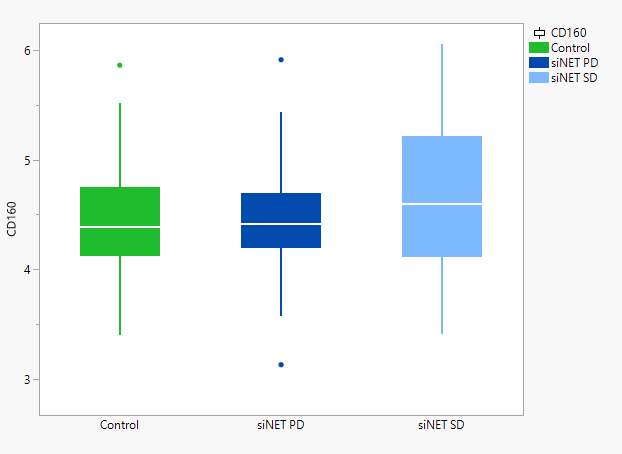


CD160: CD160 antigen; PD: Progressive Disease; SD: Stable Disease; siNET: small-intestinal NET. Control group: 143 individuals, siNET SD: 50 patients, siNET PD: 65 patients. Descriptive statistics: Mean (SD) [95% Confidence Interval]: control group: 4.44 (0.45) [4.37 – 4.52]; siNET PD: 4.45 (0.44) [4.34 – 4.56]; siNET SD: 4.61 (0.62) [4.44 – 4.79].

Figure S2. ROC curves for CgA, CPE, WISP-1 and CD160


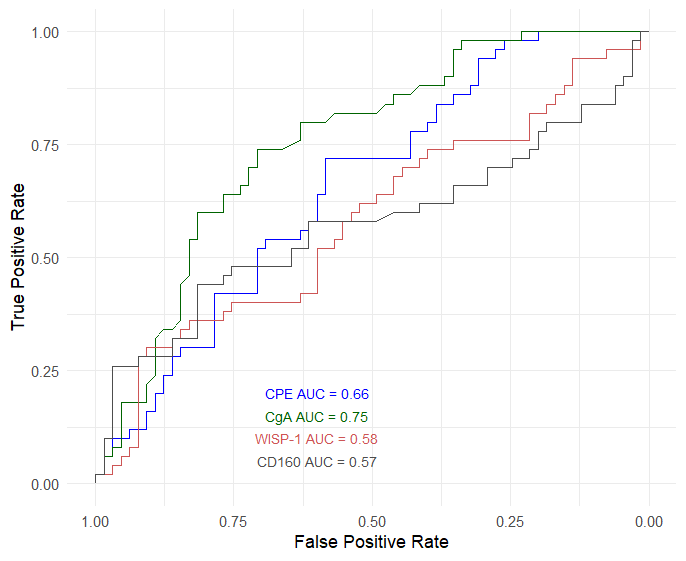


AUC: Area Under the Curve; CD160: CD160 antigen; CgA: Chromogranin A; CPE: Carboxypeptidase E; PD: progressive; ROC: Receiver Operating Characteristic curve; SD: stable disease; siNET: small-intestinal NET; WISP-1: WNT1-inducible-signaling pathway protein 1.

Logistic Regression model (R, glm) was used to predict between siNET SD and siNET PD for each biomarker independently. ROC curves were generated with the R package *pROC*. R:  [https://www.R-project.org/](https://www.r-project.org/)
